# Frequent IgE recognition of *Blomia tropicalis* allergen molecules in asthmatic children and young adults in equatorial Africa

**DOI:** 10.3389/fimmu.2023.1133935

**Published:** 2023-06-02

**Authors:** Gabrielle Pauli, Carole Wurmser, Antoine Roos, Cosme Kokou, Huey-Jy Huang, Nishelle D’souza, Christian Lupinek, Josefina Zakzuk, Ronald Regino, Nathalie Acevedo, Luis Caraballo, Susanne Vrtala, Rudolf Valenta

**Affiliations:** ^1^ Faculty of Medicine, University Louis Pasteur, Strasbourg, France; ^2^ Hôpital Albert Schweitzer, Lambaréné, Gabon; ^3^ Division of Immunopathology, Department of Pathophysiology and Allergy Research, Center for Pathophysiology, Infectiology and Immunology, Medical University of Vienna, Vienna, Austria; ^4^ Institute for Immunological Research, Universidad de Cartagena, Cartagena, Colombia; ^5^ National Research Center, Institute of Immunology FMBA of Russia, Moscow, Russia; ^6^ Laboratory for Immunopathology, Department of Clinical Immunology and Allergy, Sechenov First Moscow State Medical University, Moscow, Russia; ^7^ Karl Landsteiner University of Health Sciences, Krems, Austria

**Keywords:** allergy, allergens, asthma, equatorial Africa, allergen microarray, *Blomia tropicalis*, *Dermatophagoides*

## Abstract

**Background:**

Asthma is not well investigated in equatorial Africa and little is known about the disease-associated allergen molecules recognized by IgE from patients in this area. The aim was to study the molecular IgE sensitization profile of asthmatic children and young adults in a semi-rural area (Lambaréné) of an equatorial African country (Gabon), to identify the most important allergen molecules associated with allergic asthma in equatorial Africa.

**Methods:**

Fifty-nine asthmatic patients, mainly children and few young adults, were studied by skin prick testing to *Dermatophagoides pteronyssinus* (Der p), *D. farinae* (Der f), cat, dog, cockroach, grass, Alternaria and peanut. Sera were obtained from a subset of 35 patients, 32 with positive and 3 with negative skin reaction to Der p and tested for IgE reactivity to 176 allergen molecules from different allergen sources by ImmunoCAP ISAC microarray technology and to seven recombinant *Blomia tropicalis* (Blo t) allergens by IgE dot blot assay.

**Results:**

Thirty-three of the 59 patients (56%) were sensitized to Der p and 23 of them (39%) were also sensitized to other allergen sources, whereas 9 patients (15%) were only sensitized to allergen sources other than Der p. IgE serology analyses (n=35) showed high IgE-binding frequencies to the Blo t allergens Blo t 5 (43%), Blo t 21 (43%) and Blo t 2 (40%), whereas the Der p allergens rDer p 2, rDer p 21 and rDer p 5 (34%, 29% and 26%) were less frequently recognized. Only few patients showed IgE reactivity to allergens from other allergen sources, except to allergens containing carbohydrate determinants (CCDs) or to wasp venom allergens (i.e., antigen 5).

**Conclusion:**

Our results thus demonstrate that IgE sensitization to mite allergens is very prevalent in asthmatics in Equatorial Africa with B. tropicalis allergen molecules representing the most important ones associated with allergic asthma.

## Introduction

Asthma is a common chronic respiratory disease and an increasing health problem worldwide (1). The prevalence of asthma is very high in developed countries, but there is evidence that in some areas of the Western world it may have plateaued (2). In developing countries the prevalence is rising but still lower than in Western Countries (3). However, it is assumed that in urban areas asthma might soon reach the prevalence rates of Western Countries, probably also due to fungal infections (4–6). Additionally, the International Study of Asthma and Allergies in childhood (ISAAC) showed that the prevalence of asthma in children varied considerably between different African countries (between 5.7% in Yaoundé, Cameroon and 19.9% in Brazzaville, Congo) (4). In Gabon, the prevalence of asthma in children aged 13-14 years was 10.2%, but most studies performed so far included only urban populations and little attention has been paid to rural populations (4). Studies from other African countries also indicated that prevalence rates of asthma were considerably higher in urban than in rural areas (7).

It has long been known that sensitization to allergen sources like house dust mites (HDM), cat, dog and grasses is a major risk factor for the development of asthma (8, 9). In tropical countries, asthma is strongly associated with sensitization to HDMs and *Blomia tropicalis* (10, 11). Few studies performed in Western and equatorial Africa in recent years pointed out that sensitization to mites and cockroaches could be a risk factor for the development of asthma in these areas (12–14). However, little is known about the mite and cockroach species responsible for sensitization in Africa and there is little information about the IgE-sensitization profiles of African asthmatic patients to individual allergen molecules. Previous studies showed that IgE-reactivity profiles to individual HDM allergens varied considerably between different populations, probably because of different mite species and/or cross-reactivity to allergens from other allergen sources (15–19). In tropical and subtropical regions, it has been shown that the mite species *B. tropicalis* is prevalent, which shows only limited IgE cross-reactivity with other mite species (20–22), but in South Africa, sensitization to HDM allergen molecules dominated (17, 18, 23). The identification of the relevant mite species/allergens in Africa is crucial for the implementation of personalized and allergen-specific treatment and prevention strategies, in particular for allergen-specific immunotherapy (24, 25). Skin prick testing (SPT) with allergen extracts cannot discriminate between genuine sensitizations to HDM or Blomia because of cross-reactivity between certain allergens. So far molecular data are only available for South Africa and Zimbabwe indicating that HDM sensitization but not sensitization to Blomia is important (17, 18, 26). No molecular sensitization profiles have yet been established for tropical African regions such as Equatorial Africa.

Allergen microarrays can be used to determine patients’ IgE sensitization profiles to a panel of individual allergens and allow to distinguish between genuine and cross-sensitization to a certain allergen source (27).

In this study, 59 children and young adults (2-18 years) from a semi-rural area of Gabon, located in Equatorial Africa, with confirmed asthma were skin prick tested with eight different allergen sources, which showed that 56% of the asthmatics were sensitized to HDMs. Sera of these patients were tested for IgE-reactivity to 176 allergens from different allergen sources Interestingly, the analysis with micro-arrayed allergen molecules revealed that asthmatics from Equatorial Africa were mainly sensitized to allergens from *Blomia tropicalis* but less to *Dermatophagoides* and their IgE-reactivity profiles were different from those found in the Western world and in other geographic/climate areas in Africa. Thus this is the first study demonstrating that genuine sensitization against *Blomia tropicalis* is responsible for allergic asthma in Africa.

## Methods

### Study population

**Figure 1:**
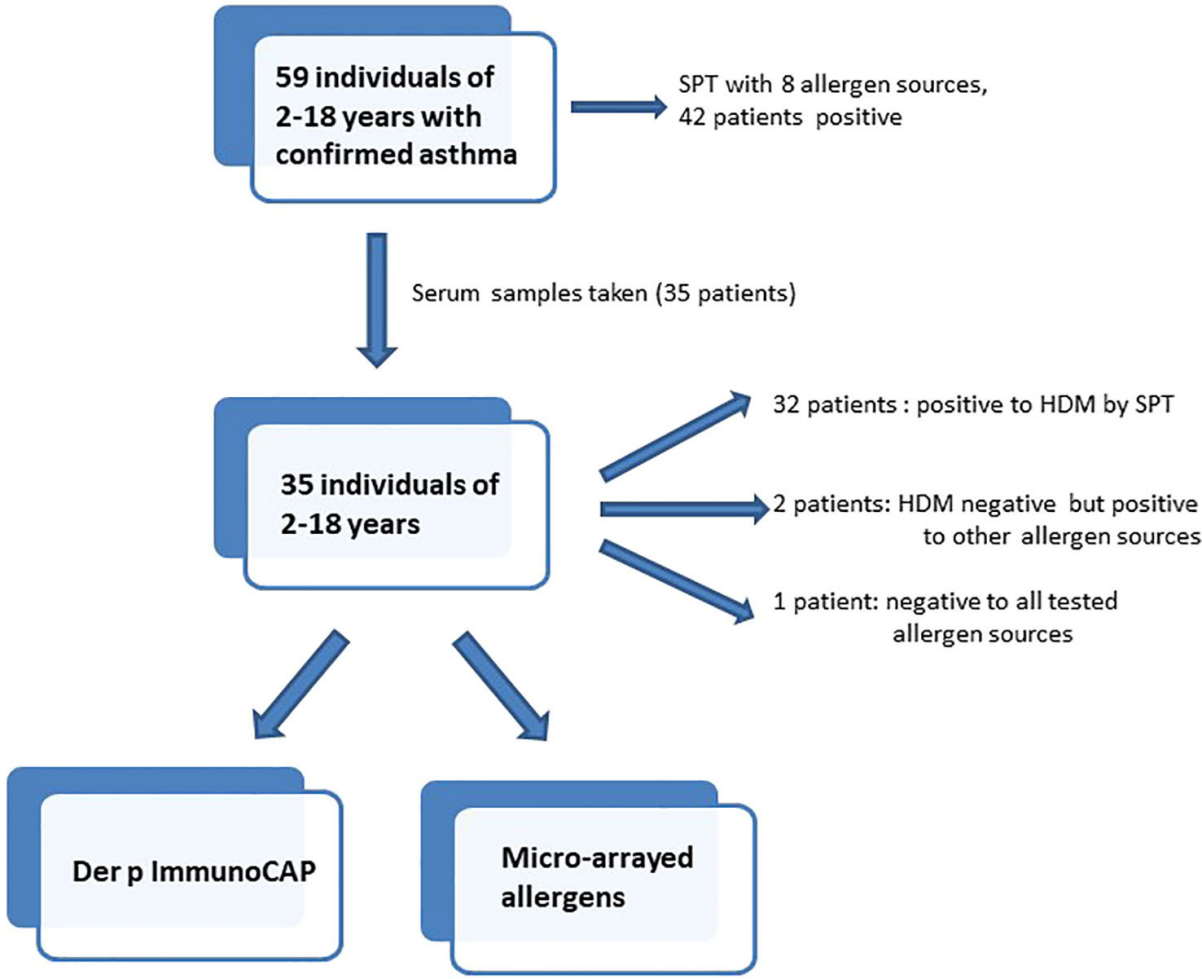
Flowchart of investigations in patients included in the study.

Fifty-nine children and young adults at the age of 2-18 with confirmed asthma were recruited consecutively from the area of Lambaréné, district of Moyen Ogooué (Gabon). Asthma symptoms were confirmed by functional investigations (FEV1 measurements and reversibility after bronchodilator (except in 18 cases when the children did not perform the test because of lack of collaboration due to low age), oxygen saturation measurements). Severity of asthma was classified according to clinical criteria according to GINA recommendations. All participants were tested by skin prick testing (SPT) to 8 different allergen sources: 2 house dust mite extracts (*Dermatophagoides pteronyssinus* and *Dermatophagoides farinae*), cat and dog extracts, cockroach, grass pollen extract, *Alternaria* and peanut extracts (Alk-Abello, Horsholm, Denmark). Sera could be obtained from 35 of the individuals, 32 of them had perennial symptoms and positive skin prick test reactions to *Dermatophagoides* extracts, the other 3 (#33-35) were negative to *Dermatophagoides* (**Table 1A**, **Figure 1**). Written informed consent was obtained from the parents of the participants. Specific IgE antibodies to *Dermatophagoides* were quantified by ImmunoCAP (d1 ImmunoCAP, ThermoFisher/Phadia, Uppsala, Sweden). IgE reactivity to a panel of purified mite allergens as well as to allergens from different allergen sources was analyzed with a customized version of the ImmunoCAP ISAC microarray (MedALL allergen-chip) (Thermo Fisher Scientific, Uppsala, Sweden) (28). **Table 1** summarizes the demographic and clinical data of the patients. **Table 2** shows the classification of the asthma severity. The study was approved by the Comité d’Ethique Institutionnel of the research center of Medical research of Lambaréné (Cermel) (CEI-MRU 01/2014) and by the Ethics Committee of the Medical University of Vienna (EK 1641/2014).

**Table 1:** Demographic and clinical characterization of the study population.

A: Demographic and clinical characterization of the 35 patients for whom IgE serology was done																
						Asthma	Der p				skin prick test (mm)					
		Sex		Age	Size	severity	spec. IgE	Histamine		Cat	Dog	Der p	Der f	grass	peanut	cockroach
1		F		4	107	2	12.8	5		0	0	6	4	0	0	0
2		M		12	144	1	4.6	6		0	0	3	3	0	0	0
3		F		15	170	1	1.1	5		0	3	3	3	0	0	ND
4		M		11	151	1	61.0	5		0	0	5.5	5	0	0	0
5		M		7	120	1	2.2	4		0	0	3	0	0	0	0
6		M		11	146	1	0.1	6		0	0	3	0	0	0	ND
7		F		7	133	1	92.5	6		0	0	7	2	0	0	0
8		F		16	165	2	7.7	5		0	0	3	3	0	0	ND
9		M		10	157	1	52.5	6		0	0	4	3	0	0	0
10		M		13	182	1	1.6	5		0	0	5	4	0	0	0
11		M		13	154	4	>100	6		3	0	6	7	2	0	3
12		F		11	163	1	0.1	8		0	3	3	0	0	0	0
13		M		11	158	3	15.2	6		0	4	4	6	0	4	0
14		M		16	173	1	43.6	8		5	0	7	7	0	5	4
15		M		10	137	2	>100	6		0	0	8	7	0	0	4
16		M		6	116	1	>100	6		0	0	4	4	3	0	0
17		M		4	112	1	3.3	5		0	0	4	4	0	0	3
18		F		14	162	1	0.3	5		0	0	3	3	0	0	3
19		F		10	160	1	0.0	4		3	0	3	3	3	3	0
20		M		9	138	1	0.3	6		0	3	4	3	0	0	0
21		M		4	108	1	0.1	5		0	0	3	0	0	3	0
22		F		3	109	1	0.0	4		2	0	0	2	2	2	0
23		M		13	153	2	33.8	8		0	0	6	6	0	0	5
24		F		9	134	1	0.1	7		2	0	2	2	2	2	3
25		M		2	86	1	0.0	5		0	0	3	3	0	4	ND
26		F		12	150	1	0.0	5		4	2	3	2	0	3	0
27		M		17	180	2	6.2	6		3	0	3	5	0	0	3
28		F		7	131	1	33.5	5		3	3	4	3	0	0	3
29		M		18	181	1	0.4	8		0	0	0	4	0	4	3
30		F		15	162	2	0.0	5		3	3	3	3	0	0	ND
31		M		14	164	1	67.5	7		5	9	5	5	0	0	3
32		M		6	130	1	17.3	5		2	0	2	3	0	0	4
33		F		5	ND	1	0.4	6		0	0	0	0	0	0	0
34		M		12	151	1	0.5	8		0	0	0	0	4	0	4
35		M		4	100	1	0.0	3		0	0	0	0	3	0	ND

B: Demographic and clinical characterization of the remaining patients without IgE serology																
						Asthma				skin prick test (mm)						
	Sex		Age		Size	severity	Histamine		Cat	Dog	Der p	Der f	grass	peanut	cockroach	Alternaria
36	F		17		162	2	6		0	0	0	0	0	0	ND	ND
37	M		14		169	2	6		0	0	0	0	0	0	ND	ND
38	M		5		111	2	5		0	0	0	0	0	0	ND	ND
39	F		11		143	2	6		0	0	0	0	0	0	0	0
40	M		2		91	1	8		0	0	0	0	0	0	ND	ND
41	F		6		112	1	3		0	0	0	0	0	0	0	0
42	F		6		126	2	5		0	3	0	0	0	3	0	0
43	F		5		ND	1	6		0	0	0	0	0	0	3	3
44	F		5		116	1	5		0	0	0	0	0	0	0	0
45	M		3		ND	1	5		0	0	0	0	0	0	0	0
46	M		7		116	1	5		0	0	0	0	0	0	0	0
47	F		5		116	1	6		0	0	0	0	0	0	0	0
48	M		2		93	2	4		0	0	0	0	0	0	0	0
49	F		17		156	3	4		0	0	0	0	3	0	0	0
50	M		11		145	1	5		0	0	0	0	0	0	0	0
51	F		2		94	2	5		0	0	0	0	0	0	0	0
52	F		6		121	1	3		2	0	0	0	2	0	0	0
53	F		5		110	2	5		0	0	0	3	3	0	3	0
54	M		7		123	1	6		0	0	0	0	0	0	0	0
55	M		2		ND	3	6		0	0	0	0	0	0	0	0
56	M		16		175	4	5		0	0	0	0	0	0	0	0
57	F		7		123	2	8		0	3	0	0	0	0	3	3
58	M		11		151	1	8		0	0	0	0	0	0	4	0
59	F		6		126	1	6		0	6	0	0	3	0	0	0

Asthma severity: 1, intermittent; 2, persistent mild; 3, persistent moderate; 4, persistent severe.

Der p spec. IgE: IgE antibodies to *D. pteronyssinus* in kUA/l.

ND, not determined.

**Table 2:** Overview of asthmatic children and young adults according to type and severity of asthma.

A, all 59 patients		
Asthma severity	n=59	age range (median)
intermittent	39 (66%)	2-18 (7)
Persistent mild	15 (25%)	2-17 (10)
Persistent moderate	3 (5%)	2-17 (9)
Persistent severe	2 (3%)	14-16 (15)

B, 35 patients of the study with serology		
Asthma severity	n=35	age range (median)
intermittent	27 (77%)	2-18 (10)
Persistent mild	6 (17%)	4-17 (14)
Persistent moderate	1 (3%)	9
Persistent severe	1 (3%)	14

### Skin prick testing

SPT were performed on the forearm, in the absence of antihistamine intake for at least one week (Alk-Abello, Horsholm, Denmark). The test was considered positive if the greatest diameter of the wheal was >3 mm or >50% of the greatest diameter of the positive control (histamine solution at 1/1000) (29). Each positive test was recorded in mm, by measuring the greatest diameter of the wheal (**Table 1**). Erythema could not be measured according to the black color of the skin.

### Quantitative IgE ImmunoCAP measurements

Specific IgE antibodies to *D. pteronyssinus* (d1) (cut-off > 0.35 kU/L) were measured according to the manufacturer’s instructions on an ImmunoCAP 100 instrument (Thermo Fisher Scientific, Uppsala, Sweden).

### Micro-array assay

A customized version of the ImmunoCAP ISAC microarray (MedALL allergen-chip) (Thermo Fisher Scientific, Uppsala, Sweden) for measuring allergen-specific IgE to 176 allergens from different allergen sources was used (25). For comparing micro-array results with skin test results performed with allergen extracts, allergens indicative for *D. pteronyssinus* sensitization were: nDer p 1, rDer p 2, rDer p 4, rDer p 5, rDer p 7, rDer p 10, rDer p 11, rDer p 14, rDer p 15, rDer p 18, rDer p 21, rDer p 23 and rDer p 37; allergens indicative for grass pollen sensitization were: rPhl p 1, rPhl p 2, nPhl p 4, rPhl p 5b, rPhl p 6, rPhl p 7, rPhl p 11, rPhl p 12; allergens indicative for cockroach sensitization were: rBla g 1, rBla g 2, rBla g 5, nBla g 7; allergens indicative for cat sensitization were: rFel d 1, nFel d 2, rFel d 4; allergens indicative for dog sensitization were: rCan f 1, rCan f 2, nCan f 3, rCan f 4, rCan f 5, rCan f 6; and allergens indicative for peanut sensitization were: Ara h 1, rAra h 2, Ara h 3, nAra h 6, rAra h 8, rAra h 9. Detection of IgE reactivity was performed as described (25) and levels of allergen-specific IgE antibodies were reported in ISAC Standardized Units (ISU) with a cut-off of 0.1 ISU.

### Purified recombinant *B. tropicalis* allergens

Recombinant Blo t 5, Blo t 8, Blo t 10 and Blo t 13 were expressed in *E. coli* strain BL21 (DE3) and purified as described (30–33). Blo t 12.0101 was expressed in *E. coli* strain Origami (DE3) under native conditions (34). Blo t 2.0104 was synthesized as a codon optimized sequence based on the gbABG76185 entry and sub-cloned into pET45b+ (Genscript, Piscataway, USA). Protein expression was done in Origami (DE3) strain as reported elsewhere (30). Blo t 2.0104 was obtained by lysing the cells and purified using nickel-nitrilotriacetic acid (Ni-NTA) resin under native conditions following recommendations for 6-His tagged proteins produced in *E. coli* (QiaExpressionist, Qiagen, Hilden, Germany). Blo t 21.0101 (gbAAX34047.1) coding sequence was amplified from a cDNA library of *B. tropicalis* and cloned into pET100 plasmid. Protein expression was performed in *E. coli* BL21 DE3 cells. To obtain a soluble product, cells were resuspended in 50 mM Tris-HCl and 30 mM NaCl pH 7.8, lysed with an ultrasonic dismembrator FB 705 (Fisher Scientific, Pittsburgh PA, USA) and centrifuged at 5000 rpm for 30 minutes at 4°C. The lysate was then incubated with Ni-NTA and purified under native conditions. Protein concentration was determined by densitometry using a BSA standard curve.

### IgE dot blotting

Two microliter aliquots of the *Blomia tropicalis* allergens, rBlo t 2, rBlo t 8, rBlo t 10, rBlo t 12, rBlo t 13 and rBlo t 21 (0.2 mg/ml) and, for control purposes, BSA were dotted onto nitrocellulose membrane strips (Schleicher & Schuell, Dassel, Germany). Nitrocellulose strips containing the dot-blotted proteins were blocked in buffer A (40mM Na_2_HPO_4_, 0.6mM NaH_2_PO_4_, pH 7.5, 0.5% Tween 20, 0.5% [w/v] BSA, 0.05% [w/v] NaN_3_) and incubated with sera (1:10 in buffer A) from the asthmatic patients and from one non-allergic individual. Bound IgE antibodies were detected with ^125^I-labeled anti-human IgE antibodies (Demeditec Diagnostics, Kiel, Germany) and visualized by autoradiography (Kodak film).

## Results

### Clinical classification of asthma in Gabonese children and young adults

The main demographic and clinical characteristics of the asthmatic children and young adults are summarized in **Tables 1A, B**. The mean age of the whole population (n=59) was 9 years (2–18), 11 were less than 5 years and the gender ratio (girl/boy) was 26/33. Aperiodic rhinitis was found in 76% of the participants. Most of the individuals were breast fed (80%) with a mean duration of 12.5 months. Even though 60% of the patients with functional investigations had lung obstruction (FEV1 less than 80%), the clinical classification resulted only in a small number of patients with severe and persistent asthma (persistent moderate, 5%, persistent severe, 3%) (**Table 2A**).

### Skin prick testing indicates that most of the asthmatic patients are sensitized to HDMs

**Figure 2:**
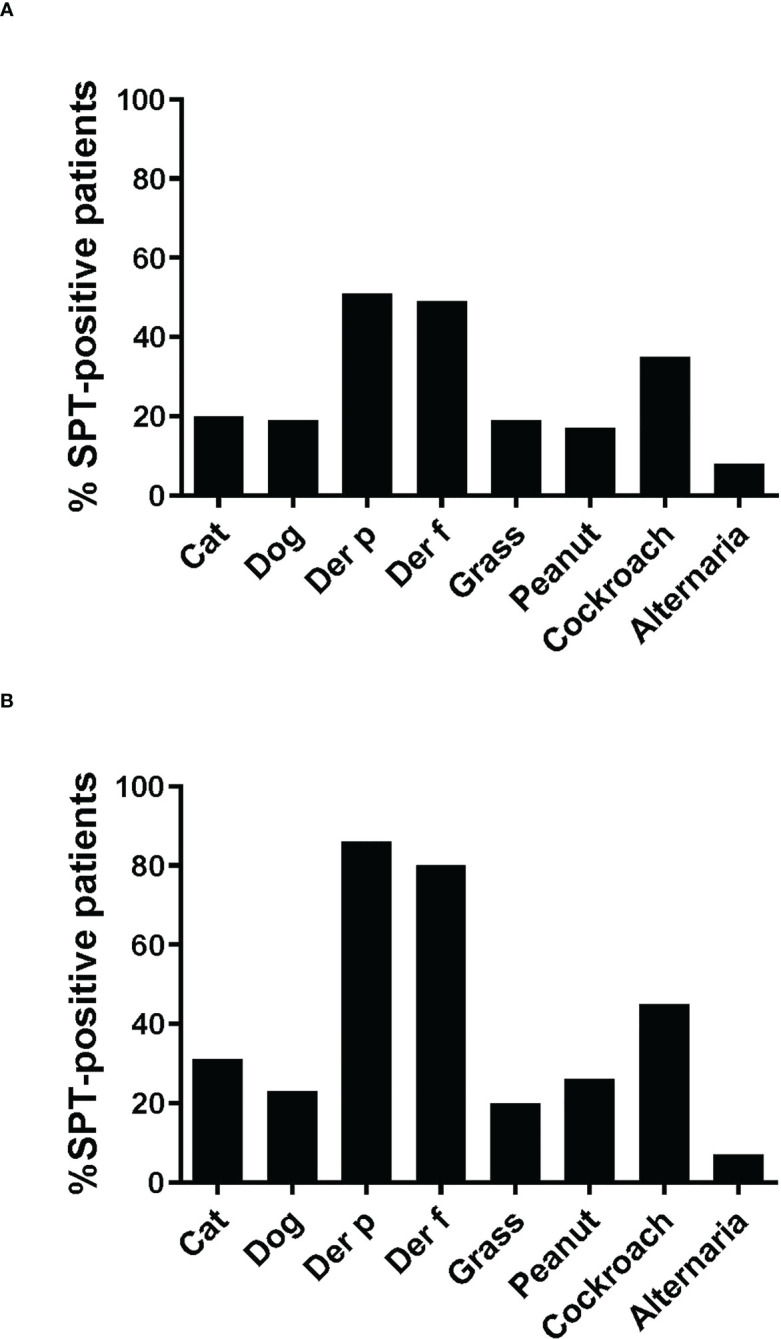
Frequencies of skin prick reactions to different allergen extracts. Frequencies of reactivity (y-axes) to cat extract, dog extract, *D. pteronyssinus* extract, *D. farinae* extract, grass pollen extract and peanut extract cockroach extract and Alternaria extract (x-axes) are shown **(A)**, for all 59 patients, **(B)**, for the 35 patients included in the study. For cockroach extract and Alternaria extract, frequencies of reactivity are shown **(A)**, for the 49 patients and **(B)**, for the 29 patients tested with these extracts.

The frequencies of skin prick reactions to eight different allergen sources are shown in **Figure 2A** for the 59 asthmatics. Ten of the patients were not tested with cockroach and *Alternaria* extracts, as these extracts were not available at the beginning of the study (**Tables 1A, B**). Thirty-three out of the 59 individuals were sensitized to at least one of the *Dermatophagoides* species (56%), four of them were only sensitized to *D. pteronyssinus*, three only to *D. farinae* (**Tables 1A, B**, **Figure 2A**). Twenty-three of the 33 HDM sensitized patients also showed a positive response to one or more other allergen sources, the other 10 patients were exclusively sensitized to HDMs. Nine patients were only sensitized to allergen sources other than HDMs (15%), the remaining 17 patients did not show a positive response to any of the tested allergen sources (**Table 1**). Thirty-five % of the patients were sensitized to cockroach, but only one patient was monosensitized. The frequencies of positive SPT to the other tested allergen extracts were considerably lower (cat: 20%, dog: 19%; grass: 19%; peanut: 17% and Alternaria: 8%) (**Figure 2A**). Two individuals were exclusively sensitized to grass pollen extract, but no monosensitization was found for cat, dog, peanut or *Alternaria* (**Table 1**).

### Asthmatics from Gabon show high IgE-reactivity to *Blomia tropicalis* as well as *Dermatophagoides* allergens

Since more than 50% of the asthmatic Gabonese patients were sensitized to HDMs, we were interested to study the IgE-reactivity profile of them to HDM allergens. For this purpose, serum samples were taken from a subgroup of 32 patients with sensitization to HDMs, and for control purposes, from 2 patients without cutaneous reactions to HDMs but with sensitization to other allergen sources as well as from one individual without positive skin reaction to any of the tested allergen sources (**Figure 1**). **Table 1A** shows the demographic and clinical characteristics of these 35 patients. The group of HDM sensitized patients was comparable to the whole population regarding type and severity of asthma (**Table 2B**). The selected subgroup of 35 individuals also reacted more frequently to cockroach (45% versus 35%), peanut (26% versus 17%) and cat (31% versus 20%) than the total population, whereas the frequencies of skin reactions were comparable for dog, *Alternaria* and grass between the total population and the subgroup of HDM sensitized patients (**Figure 2**). Sera from the subgroup of 35 asthmatic patients were tested by ImmunoCAP (d1) for the presence of *D. pteronyssinus* specific IgE antibodies. Four patients, who showed only weak positive cutaneous reactions to HDMs (diameter, 3mm) were negative for *D. pteronyssinus* in the ImmunoCAP (**Table 1A**, Der p spec. IgE). Further four patients with low *D. pteronyssinus* specific IgE levels (0.1 kUA/l) also had only tiny wheal diameters after testing with HDMs (< 3mm) (**Table 1A**).

**Figure 3:**
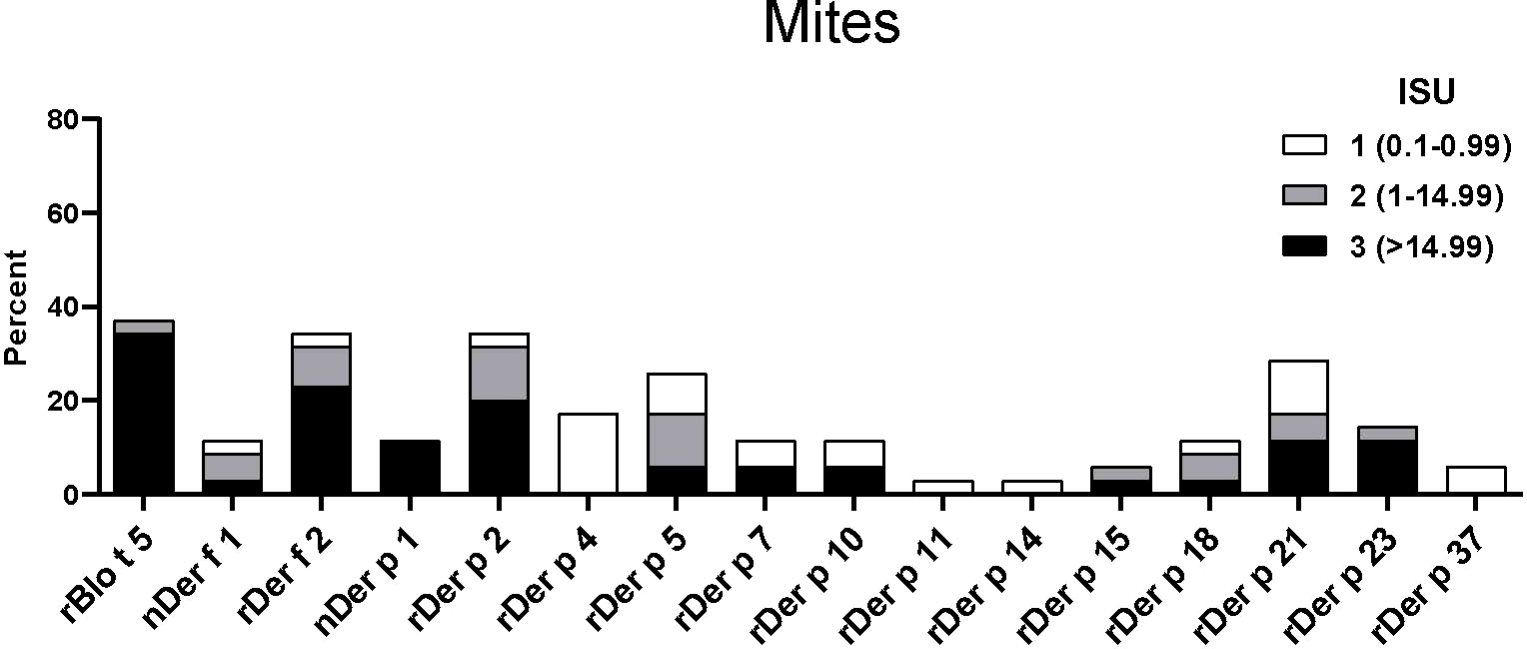
Frequencies and intensities of IgE reactivity to individual mite allergens. Frequencies of IgE reactivity and specific IgE levels (white, ISU = 0.1-0.99; grey, ISU 1-14.99; black, ISU > 14.99) (y-axis) to 16 mite allergens (x-axis) determined by micro-array analysis are shown for the 35 patients of the study.

The IgE reactivity profile of the subgroup of 35 patients to HDM allergens was determined by microarray technology (MedALL chip). Interestingly, the highest IgE-binding frequency was found for the *Blomia tropicalis* allergen rBlo t 5 (37%) and 12 of the 13 patients with IgE reactivity to rBlo t 5 had high IgE-levels (>15 ISU, median IgE level: 65.89) to this allergen (**Figure 3**). By contrast, the median IgE level to Der p 5, the HDM allergen homologous to Blo t 5, was much lower (i.e., 1.31 ISU). The most frequently recognized *D. pteronyssinus* allergens were rDer p 2, rDer p 21 and rDer p 5 (34%, 29% and 26%, respectively), whereas nDer p 1 and rDer p 23, which are known as major allergens in European populations, were only recognized by 11% and 14% of the patients. However, almost all of these patients had IgE-levels >15 ISU to nDer p 1 and/or rDer p 23 and the median IgE levels of nDer p 1 (21.65 ISU) and rDer p 23 (30.54 ISU) were comparable to that of rDer p 2 (26 ISU). The IgE-binding frequencies to other *D. pteronyssinus* allergens varied between 3% (rDer p 11 and rDer p 14) and 17% (rDer p 4) (**Figure 3**).

### Asthmatics from Gabon frequently show IgE-reactivity to cross-reactive carbohydrate determinants (CCDs) and to antigen 5 from wasp

**Figure 4:**
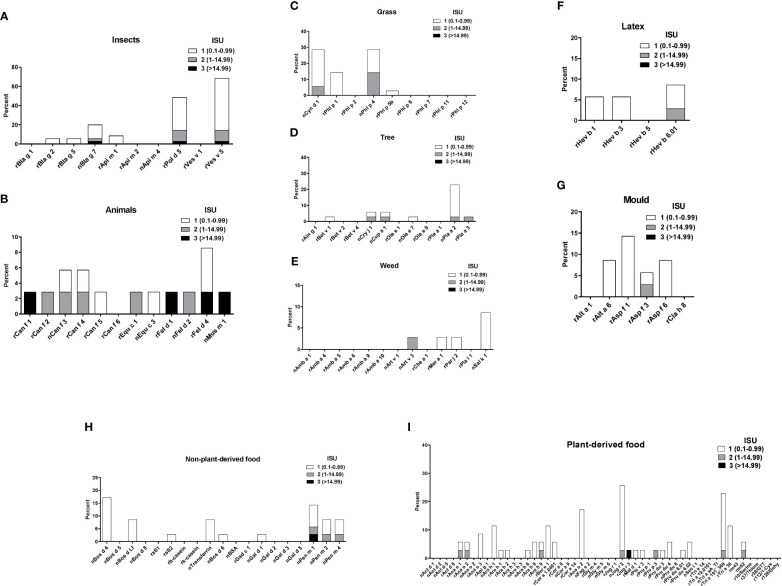
Frequencies and intensities of IgE reactivity to individual allergens from different allergen sources. Frequencies of IgE reactivity and specific IgE levels (white, ISU = 0.1-0.99; grey, ISU 1-14.99; black, ISU > 14.99) (y-axis) to individual allergens from **(A)**, insects, **(B)**, animals, **(C)**, grasses, **(D)**, trees, **(E)** weeds, **(F)**, latex, **(G)**, mould, **(H)**, non-plant derived food and **(I)**, plant derived food (x-axis) as determined by micro-array are shown for the 35 patients with IgE serology.

Sera from the 35 asthmatics also showed IgE-reactivity to several allergens from different allergen sources in ImmunoCAP ISAC. No significant IgE sensitizations were found for honeybee venom allergens or the wasp allergen, rVes v 1 but 69% of the patients had IgE to rVes v 5 and 54% to the Ves v 5-cross-reactive allergen, rPol d 5 (**Figure 4A**). Interestingly, only few patients showed IgE-reactivity to the major cockroach allergens, rBla g 2 and rBla g 5, but more than 20% of the patients reacted to the highly-cross-reactive cockroach tropomyosin, nBla g 7 (**Figure 4A**). Similar IgE-binding frequencies were found for tropomyosins from HDM (rDer p 10, 11%) (**Figure 3**) and shrimp (nPen m 1, 14%) (**Figure 4H**) and several patients had high IgE-levels (>15 ISU) to the tropomyosins (**Figure 4**). Only one patient with a mixed African/European ethnicity (patient #31) showed relevant IgE reactivity to allergens from animals (**Figure 4B**). Only few of the asthmatics were sensitized to the major grass pollen allergens, rPhl p 1 and rPhl p 5 and none of the patients were sensitized to rPhl p 2, 6, 7, 11 or 12 (**Figure 4C**). On the other hand, high IgE-binding frequencies (30%) were found for allergens containing cross-reactive carbohydrate determinants (CCDs) (i.e., nCyn d 1, nPhl p 4) and these patients also showed IgE-reactivity to other glycosylated allergens (nPla a 2, nJug r 2) (**Figures 4D–I**). No significant IgE sensitizations were found for tree pollen allergens of the European tree families *Betulaceae* and *Oleaceae* or for weed allergens (**Figures 4D, E**). Only few patients showed low IgE-reactivity to allergens from latex and moulds (**Figures 4F, G**). Beside the shrimp tropomyosin, nPen m 1, also nPen m 2 and 4 were recognized by some of the asthmatics, whereas no relevant IgE sensitization was found to milk or egg allergens (**Figure 4H**). Although peanuts are eaten regularly in Gabon, only few patients had low IgE-levels to peanut allergens. Low levels of IgE-reactivity were also detected to allergens from other nuts and to wheat allergens (**Figure 4I**).

### Comparison of cutaneous tests and IgE-reactivity to individual allergens

**Figure 5:**
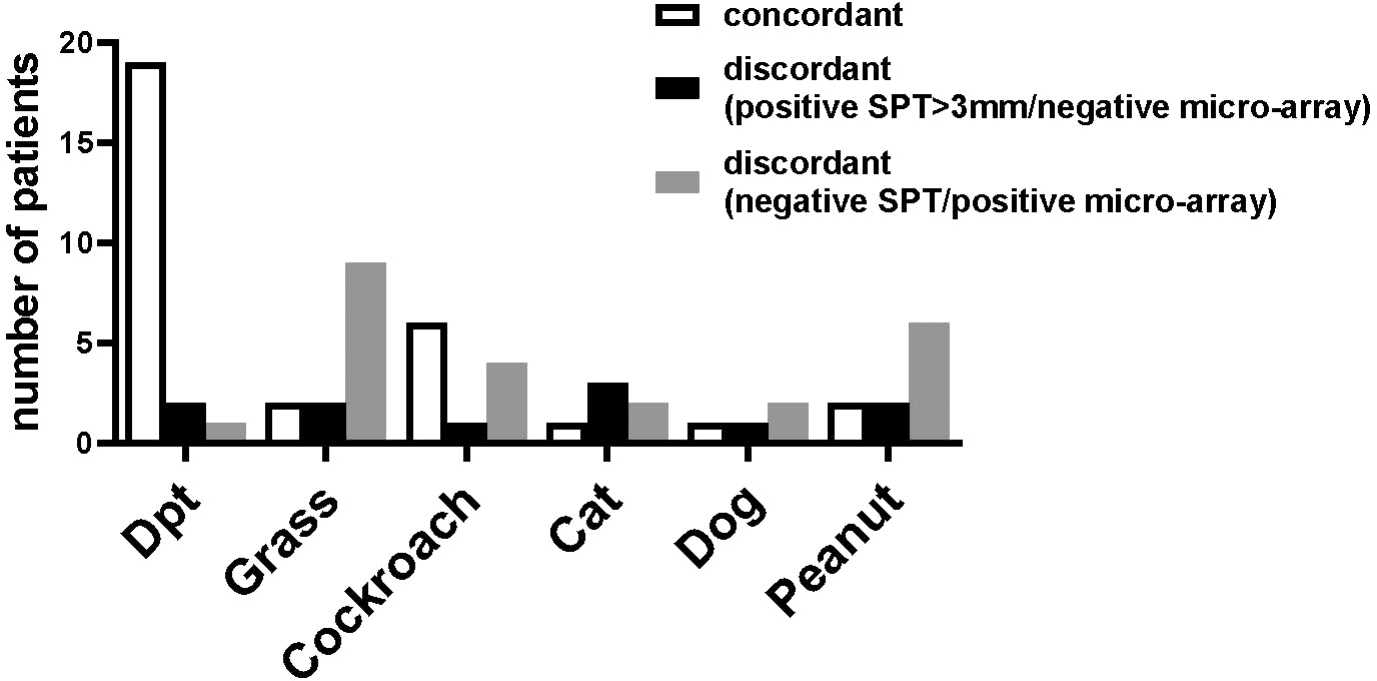
Concordances and discordances between skin prick tests and micro-array analysis. The number of patients (y-axis) with concordant results (left bar) or discordant results in skin prick tests and micro-array (central bar, positive SPT >3mm/negative ISAC, right bar, negative SPT/positive micro-array) for *D. pteronyssinus*, grass, cockroach, cat, dog and peanut (x-axis) are shown for the 35 patients of the study and for cockroach for the 29 patients tested by SPT.

The results of the SPT and the ImmunoCAP ISAC were not concordant in all patients and the number of patients with discordant results varied according to the allergen source (**Figure 5**). Most patients (19 patients) with positive SPT to *D. pteronyssinus* extracts also showed IgE reactivity to recombinant HDM allergens, whereas for other allergen sources this number was considerably lower (**Figure 5**, left columns). For all tested allergen sources, several patients were found with positive cutaneous tests but negative test to the individual allergens; however, this number was rather low (up to 3 patients), if only SPT results >3 mm were considered positive (**Figure 5**, central columns). In contrast, some patients with negative SPTs were positive in ImmunoCAP ISAC, in particular for grass and peanut allergens (**Figure 5**, right column). The IgE-values to the peanut allergens were however very low (<0.3 ISU) and in the case of grass pollen allergens, the CCD-containing allergens (e.g., nPhl p 4) were recognized which have low clinical relevance (**Figure 5**, right columns).

### rBlo t 2, rBlo t 5 and rBlo t 21 were the most frequently recognized *Blomia tropicalis* allergens in asthmatics that were diagnosed as positive to a *D. pteronyssinus* extract

**Figure 6:**
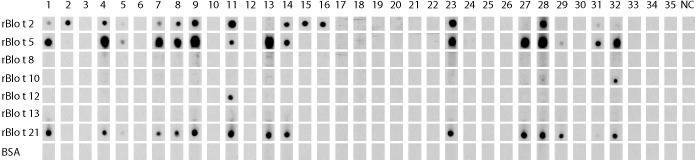
IgE-reactivity to *Blomia tropicalis* allergens. Dot blotted *Blomia tropicalis* allergens (rBlo t 2, rBlo t 5, rBlo t 8, rBlo t 10, rBlo t 12, rBlo t 13 and rBlo t 21) and BSA were tested for IgE-reactivity with sera from the 35 asthmatic patients of the study (1-35) and serum from a non-allergic individual (NC).

When the IgE reactivity profile of the subgroup of 32 Der p-positive asthmatics was determined by microarray assay, the highest IgE-binding frequency was found for the *Blomia tropicalis* allergen rBlo t 5 (37%) (**Figure 3**). Therefore, sera from the 32 Der p-positive and three Der p-negative patients were further tested for IgE-reactivity to *Blomia tropicalis* allergens which were not present on the allergen chip (rBlo t 2, rBlo t 8, rBlo t 10, rBlo t 12, rBlo t 13 and rBlo t 21) by dot blot assay (**Figure 6**). The rBlo t 5-specific IgE-reactivity (43% in dot blot) correlated well with the allergen chip results, only two patients with very low IgE-reactivity to rBlo t 5 in the dot blot (patients 5 and 29) were negative in the microarray assay (**Figure 6**). High IgE-binding frequencies were also found for rBlo t 21 (43%) and rBlo t 2 (40%), whereas the other tested allergens were only recognized by few patients (rBlo t 10, 6%; rBlo t 12, 3%). None of the patients showed IgE-reactivity to rBlo t 8, rBlo t 13 and the negative control protein, BSA (**Figure 6**).

### Patients with severe asthma reacted more often to *B. tropicalis* and HDM allergens than patients with mild asthma

**Figure 7:**
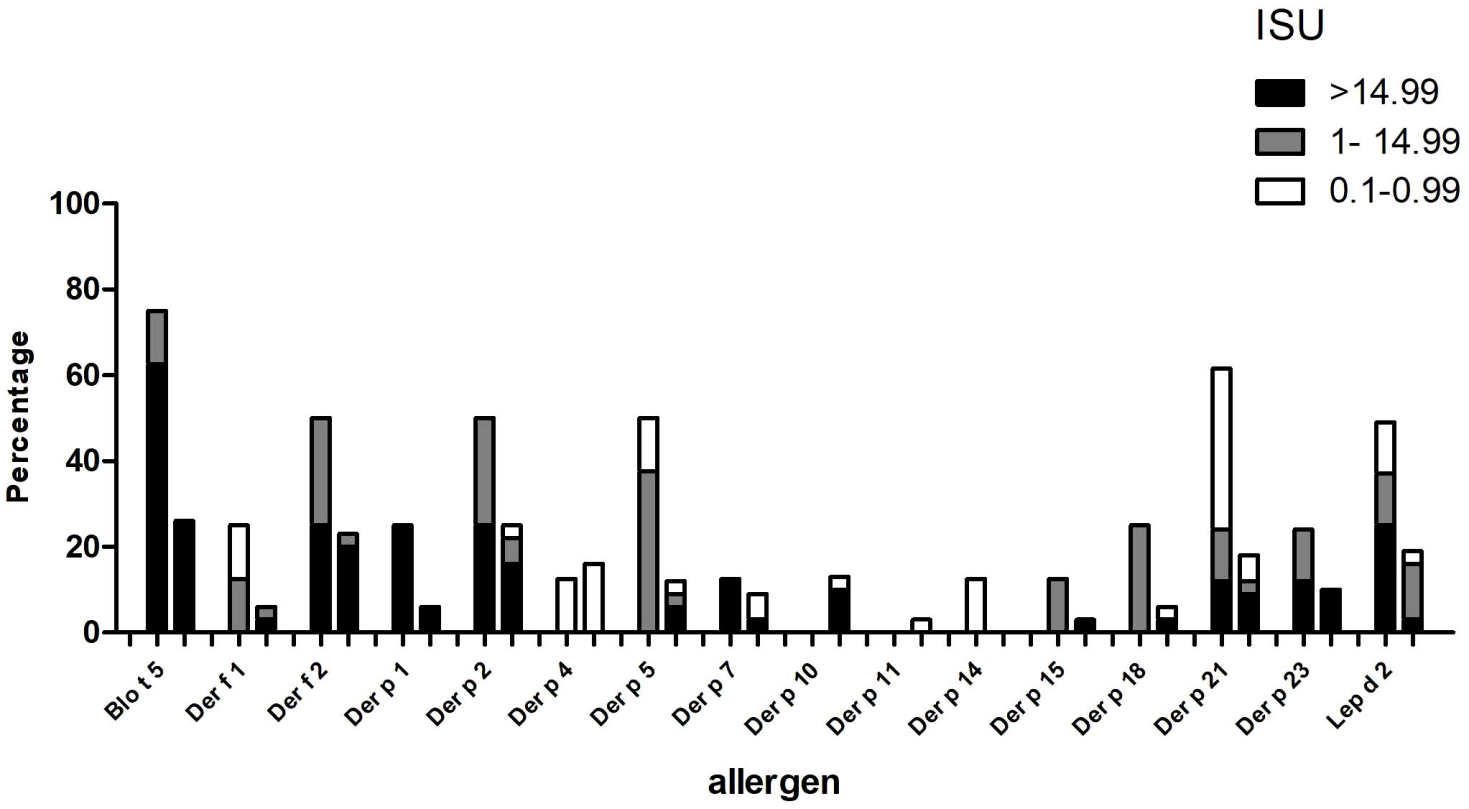
Frequencies of IgE sensitization and IgE levels to Blo t 5 and house dust mite allergens (x-axis) (ISU = 0.1-0.99; ISU 1-14.99; ISU > 14.99) (y-axis) in patients with different asthma severity scores, 2-4 (left columns) or <2 (right columns).

When we compared patients with an asthma severity score of <2 with those with asthma severity scores of 2-4 regarding IgE binding frequency and IgE levels to Blo t 5 and HDM allergens, we found that patients with more severe asthma reacted more often to Blo t 5 and to HDM allergens than patients with a low asthma severity score (**Figure 7**). Furthermore, allergen-specific IgE levels were higher in patients with asthma scores 2-4 as compared to patients with asthma scores <2 (**Figure 7**). Due to the low number of patients (i.e., n=35), it was not possible to perform statistical analysis.

## Discussion

The International Study of Asthma and Allergies in Childhood has completed phase 3 and it has now become clear that asthma has increased in Africa (4). For instance, in the age range 0-15 years, an increase of 50% of the total number of asthma cases was observed (35). However, the focus has remained on urban populations and not many studies have paid attention to rural populations (17). Furthermore, a systematic investigation of IgE sensitizations in different climate zones in Africa are lacking. Here we studied the IgE-sensitization profile of asthmatic children and young adults in a semi-rural area of equatorial Africa, to identify the most important allergens involved in allergic asthma in this area.

On the basis of clinical data, we observed a high rate of intermittent asthma and a rather low rate of more severe asthma (persistent and severe), which was different to functional data, which showed a high level of bronchial obstruction. However, reference values were not available for African children, the only reference values were established in American black children and showed lower values (minus 14%) in comparison to Caucasian children (36).

Seventeen of the 59 asthmatic patients did not show skin sensitivity with any of the tested allergen extracts. These patients could be sensitized to an allergen source that was not used for SPT (e.g., *B. tropicalis*), but also respiratory virus infections have been shown as important trigger factors for wheezing attacks, especially in young children (37). Thirty-three of our 59 asthmatic patients (56%) showed positive skin prick test responses to *D. pteronyssinus* and/or *D. farinae* extracts. Previous studies from Zimbabwe reported HDM sensitization rates between 53% and 67.8% in atopic populations (38, 39) and studies from tropical Africa (Nigeria and Cameroon) found a HDM-specific sensitization rate of 45% and 68.9%, respectively, in asthmatic children (12, 13). In an earlier study performed in 520 schoolchildren of the district of Lambaréné, 11% of the children were sensitized to *Dermatophagoides* species (40). A birth cohort study performed in Uganda pointed out that the most important allergen sources in sub-Saharan Africa were *Dermatophagoides* species (sensitization rate: 18%) and *Blomia tropicalis* (15%) (41).

The major aim of this study was to determine the molecular sensitization profile of asthmatic children and young adults with positive skin prick test to commercial *D. pteronyssinus* allergen extract. Interestingly, we found the highest rate of sensitization for Blo t 5 from *Blomia tropicalis* (37%) with very high IgE levels (median 65.89 ISU), which agrees with results obtained by skin prick test to commercial *B. tropicalis* allergen extract in adjacent countries with the same tropical climate with high temperatures and high humidity [Nigeria (11) Cameroun (13)]. This finding is in contrast to molecular studies performed in South Africa where sensitization to HDM allergens dominated and sensitization to Blomia allergens (i.e., Blo t 5) was rare (17, 18, 23). The differences are most likely due to different climatic conditions, since in Gabon a tropical climate like in Brazil and South Asia can be found.

It was shown that there is little IgE cross reactivity between most *B. tropicalis* and *D. pteronyssinus* allergens and in our study the IgE levels of Blo t 5-homologous allergens from *D. pteronyssinus* (rDer p 5 and rDer p 21) were much lower than the rBlo t 5 IgE levels (42). This was also supported by our dot blot results with recombinant Blo t allergens, which showed that considerably higher IgE-binding frequencies were found to rBlo t 21 than to rDer p 21, whereas for rDer p 2 and rBlo t 2 the frequency of reactivity was similar. This might be due to cross-reactivity between Der p 2 and Blo t 2, or because we used an isoform (Blo t 2.0104) that is not prevalent in the mite and might show lower IgE-reactivity (43). It must be noted that little is known about the distribution of mite species in sub-Saharan Africa, but there is evidence that *B. tropicalis* represents an important mite species also in other regions with a hot and humid climate (44, 45). Unfortunately, *B. tropicalis* extracts were not used for skin prick testing, since it was not expected by the clinical investigators that Blomia might be a genuinely sensitizing allergen source in Gabon. When the molecular analysis performed with the obtained serum samples revealed Blomia as genuinely sensitizing allergen source, it was not possible to repeat skin testing with Blomia extracts. The fact that *B. tropicalis* was the most important allergen source in this population may have different reasons. One likely possibility is that *B. tropicalis* sensitization depends very much on specific climate conditions, because this mite species can only survive at high temperatures and humidity (46). In fact, high frequencies of IgE sensitization to *B. tropicalis* allergens have been reported for several countries in South America, Asia and now, as reported by us, in Africa, which are in the vicinity of the equator (10, 20, 44). It is also quite possible that hygienic conditions play and important role (47), but sensitization to *B. tropicalis* allergens is also very common in highly industrialized areas in Asia with high standards of living (48, 49). Yet there may be subtle differences regarding IgE sensitizations of Gabon patients compared to *B tropicalis*-sensitized patients from other regions. For example Gabon patients did not recognize certain *B. tropicalis* allergens, such as rBlo t 13, which is recognized by more than 10% of the patients in South America (50). This might be caused by differences in the allergen content between mites of different continents (51).

High sensitization rates were also found for rDer p 2 (34%), whereas the allergens of group 1 were less frequently recognized (11%). However, the highest median of IgE titers were found for rDer p 23 (30.54 ISU), followed by rDer p 2 (26 ISU) and nDer p 1 (21.65 ISU), confirming that these three allergens are important for this African population. In a European study with 105 asthmatic children, a higher rate of sensitization was observed for nDer p 1, rDer p 2 and rDer p 23 (77%, 81% and 71%, respectively), but the median IgE titers were lower (52).

The majority of patients (24 out of 35) had specific IgE to rVes v 5 (antigen 5) and 19 of the 24 rVes v 5-positive patients also showed IgE-reactivity to the cross-reactive allergen, rPol d 5. Thus, the patients may have been stung by wasps or may have been sensitized to allergens which cross-react with antigen 5 (e.g., *Solenopsis africana*, which represents an endemic red fire ant in Gabon) (53). Only the two glycosylated grass pollen allergens, nCyn d 1 and nPhl p 4, were recognized by our asthmatic patients, who also showed IgE reactivity to other glycosylated pollen allergens, e.g. to nPla a 2. Similar results were observed in Central Africa and the Philippines, but it is supposed that IgE sensitizations to carbohydrate epitopes are not clinically relevant (54). It is feasible that parasitic infections could also influence the results on IgE reactivity to CCD or the IgE response to invertebrate pan-allergens such as tropomyosin (32). Therefore, further immunological investigations would be necessary to determine the primary sensitizing tropomyosin in our population.

Some discordances between skin prick tests and ImmunoCAP ISAC have been observed in our study, which might be explained by low quality of the skin prick test extracts (55), by IgE sensitization to CCD-containing plant allergens which do not elicit skin reactivity (e.g., nPhl p 4) (56) or by sensitization of the African patients to allergens which were not present on the allergen chip. Additionally, it has been shown before that living under poor hygienic conditions, childhood infections and genetic factors may contribute to a dissociation between specific IgE and skin prick test reactivity (57). Our study has three limitations: Skin testing with Blomia extract could not be repeated, the study was mainly descriptive and the number of patients studied was relatively small.

Nevertheless, this is the first study on the exact IgE sensitization profile in a semi-rural asthmatic equatorial African population and showed: i) a predominant sensitization to allergens of the tropical mite *Blomia tropicalis;* ii) much lower sensitization to *Dermatophagoides pteronyssinus* with a molecular sensitization profile which differs partly from European asthmatics; iii) low frequency of sensitization to most of the other tested allergens. Further investigations are needed to identify the relevant mite species in house dust samples and the clinically relevant mite allergens for this population, in order to improve diagnosis and immunotherapy in this area (58). Immunotherapy trials with Blomia extracts are scarce, although some effort has already been put on the development of new therapeutics for the treatment of Blomia sensitized patients (59–62). Based on our results we hypothesize now that it may be useful to select those patients for the correct form of immunotherapy, i.e. Blomia for genuine IgE sensitization to Blomia, HDM for genuine IgE sensitization to HDMs or Blomia and HDM for patients with co-sensitization.

## Data availability statement

The original contributions presented in the study are included in the article/supplementary material. Further inquiries can be directed to the corresponding author.

## Ethics statement

The study was approved by the Comité d’Ethique Institutionnel of the research center of Medical research of Lambaréné (Cermel) (CEI-MRU 01/2014) and by the Ethics Committee of the Medical University of Vienna (EK 1641/2014). Written informed consent to participate in this study was provided by the participants’ legal guardian/next of kin.

## Author contributions

GP contributed to the design of the study, interpreted the findings and wrote the manuscript. SV and RV designed the study and contributed to the interpretation of the findings, writing and revising the manuscript. CW, AR, CK, H-JH, ND’S, CL, JZ, RR, NA and LC performed the experiments, provided materials, read and revised the manuscript. All authors contributed to the article and approved the submitted version.

## References

[1] Hedlin G van Hage M. Severe asthma and allergy: mechanisms, diagnostics and treatment. J Intern Med (2012) 272:104–7. doi: 10.1111/j.1365-2796.2012.02557.x 22632711

[2] Eder W Ege MJ von Mutius E. The asthma epidemic. N Engl J Med (2006) 355:2226–35. doi: 10.1056/NEJMra054308 17124020

[3] Wjst M Boakye D. Asthma in Africa. PloS Med (2007) 4:e72. doi: 10.1371/journal.pmed.0040072 17326712 PMC1808099

[4] Ait-Khaled N Odhiambo J Pearce N Adjoh KS Maesano IA Benhabyles B. Prevalence of symptoms of asthma, rhinitis and eczema in 13- to 14-year-old children in Africa: the international study of asthma and allergies in childhood phase III Allergy. (2007) 62:247–58. doi: 10.1111/j.1398-9995.2007.01325.x 17298341

[5] Skevaki C Ngocho JS Amour C Schmid-Grendelmeier P Mmbaga BT Renz H. Epidemiology and management of asthma and atopic dermatitis in Sub-Saharan Africa. J Allergy Clin Immunol (2021) 148:1378–86. doi: 10.1016/j.jaci.2021.10.019 34715154

[6] Hadebe S Brombacher F. Environment and host-genetic determinants in early development of allergic asthma: contribution of fungi. Front Immunol (2019) 10:2696. doi: 10.3389/fimmu.2019.02696 31824491 PMC6879655

[7] Desalu OO Adeoti AO Ojuawo OB Aladesanmi AO Oguntoye MS Afolayan OJ. Urban-rural differences in the epidemiology of asthma and allergies in Nigeria: a population-based study. J Asthma Allergy (2021) 14:1389–97. doi: 10.2147/JAA.S333133 PMC8637762 34866916

[8] Lodge CJ Lowe AJ Gurrin LC Hill DJ Hosking CS Khalafzai RU. House dust mite sensitization in toddlers predicts current wheeze at age 12 years. J Allergy Clin Immunol (2011) 128:782–88. doi: 10.1016/j.jaci.2011.06.038 21820717

[9] Sanchez-Borges M Fernandez-Caldas E Thomas WR Chapman MD Lee BW Caraballo L. International consensus (ICON) on: clinical consequences of mite hypersensitivity, a global problem. World Allergy Organ J (2017) 10:14. doi: 10.1186/s40413-017-0145-4 28451053 PMC5394630

[10] Fernandez-Caldas E Baena-Cagnani CE Lopez M Patino C Neffen HE Sanchez-Medina M. Cutaneous sensitivity to six mite species in asthmatic patients from five Latin American countries. J Investig Allergol Clin Immunol (1993) 3:245–9. 8298748

[11] Alberca-Custodio RW Faustino LD Gomes E Nunes FPB de Siqueira MK Labrada A. Allergen-specific immunotherapy with liposome containing CpG-ODN in murine model of asthma relies on MyD88 signaling in dendritic cells. Front Immunol (2020) 11:692. doi: 10.3389/fimmu.2020.00692 32391011 PMC7191058

[12] Oluwole O Arinola OG Falade GA Ige MO Falusi GA Aderemi T. Allergy sensitization and asthma among 13-14 year old school children in Nigeria. Afr Health Sci (2013) 13:144–53. doi: 10.4314/ahs.v13i1.20 PMC3645088 23658581

[13] Pefura-Yone EW Mbele-Onana CL Balkissou AD Kenmegne-Noumsi EC Boulleys-Nana JR Kolontchang-Yomi BL. Perennial aeroallergens sensitisation and risk of asthma in African children and adolescents: a case-control study. J Asthma (2015) 52:571–5. doi: 10.3109/02770903.2014.995306 25494554

[14] Herrant M Loucoubar C Boufkhed S Bassene H Sarr FD Baril L. Risk factors associated with asthma, atopic dermatitis and rhinoconjunctivitis in a rural Senegalese cohort. Allergy Asthma Clin Immunol (2015) 11:24. doi: 10.1186/s13223-015-0090-0 26306096 PMC4547418

[15] Weghofer M Thomas WR Kronqvist M Mari A Purohit A Pauli G. Variability of IgE reactivity profiles among European mite allergic patients. Eur J Clin Invest (2008) 38:959–65. doi: 10.1111/j.1365-2362.2008.02048.x 19021722

[16] Hales BJ Laing IA Pearce LJ Hazell LA Mills KL Chua KY. Distinctive immunoglobulin e anti-house dust allergen-binding specificities in a tropical Australian aboriginal community. Clin Exp Allergy (2007) 37:1357−63. doi: 10.1111/j.1365-2222.2007.02786.x 17845417

[17] Mittermann I Dzoro S Gattinger P Botha M Basera W Facey-Thomas HE. Molecular IgE sensitization profiles of urban and rural children in south Africa. Pediatr Allergy Immunol (2021) 32:234–41. doi: 10.1111/pai.13377 32969537

[18] Muddaluru V Valenta R Vrtala S Schlederer T Hindley J Hickey P. Comparison of house dust mite sensitization profiles in allergic adults from Canada, Europe, south Africa and USA. Allergy (2021) 76:2177–88. doi: 10.1111/all.14749 33484161

[19] Pinheiro CS Silva ES de Andrade Belitardo EMM Pacheco LGC Aguiar E Alcantara-Neves NM. En route to personalized medicine: uncovering distinct IgE reactivity pattern to house dust mite components in Brazilian and Austrian allergic patients. Clin Transl Allergy (2021) 11:e12004. doi: 10.1002/clt2.12004 33900048 PMC8099267

[20] Rodriguez O Labrada A. Diagnostic clinical trial in children using an allergenic extract of blomia tropicalis. Allergol Immunopathol (Madr) (2000) 28:225–8. 11022269

[21] Thomas WR Hales BJ Smith W. Blomia tropicalis: more than just another source of mite allergens. Clin Exp Allergy (2003) 33:416–8. doi: 10.1046/j.1365-2222.2003.01634.x 12680854

[22] Fernandez-Caldas E Lockey RF. Blomia tropicalis, a mite whose time has come. Allergy. (2004) 59:1161–4. doi: 10.1111/j.1398-9995.2004.00727.x 15461595

[23] Van Rooyen C Van den Berg S Becker PJ Green RJ. Allergic sensitisation in south Africa: exploring regional variation in sensitisation. S Afr Med J (2020) 110:686–90. doi: 10.7196/SAMJ.2020.v110i7.14420 32880348

[24] Rodinkova VV Yuriev SD Kryvopustova MV Mokin VB Kryzhanovskyi YM Kurchenko AI. Molecular profile sensitization to house dust mites as an important aspect for predicting the efficiency of allergen immunotherapy. Front Immunol (2022) 13:848616. doi: 10.3389/fimmu.2022.848616 35392080 PMC8980548

[25] Rodriguez-Dominguez A Berings M Rohrbach A Huang HJ Curin M Gevaert P. Molecular profiling of allergen-specific antibody responses may enhance success of specific immunotherapy. J Allergy Clin Immunol (2020) 146:1097–108. doi: 10.1016/j.jaci.2020.03.029 32298697

[26] Westritschnig K Sibanda E Thomas W Auer H Aspock H Pittner G. Analysis of the sensitization profile towards allergens in central Africa. Clin Exp Allergy (2003) 33:22–7. doi: 10.1046/j.1365-2222.2003.01540.x 12534545

[27] Huang HJ Campana R Akinfenwa O Curin M Sarzsinszky E Karsonova A. Microarray-based allergy diagnosis: quo vadis? Front Immunol (2020) 11:594978. doi: 10.3389/fimmu.2020.594978 33679689 PMC7928321

[28] Lupinek C Wollmann E Baar A Banerjee S Breiteneder H Broecker BM. Advances in allergen-microarray technology for diagnosis and monitoring of allergy: the MeDALL allergen-chip. Methods. (2014) 66:106–19. doi: 10.1016/j.ymeth.2013.10.008 PMC4687054 24161540

[29] Stemeseder T Metz-Favre C de Blay F Pauli G Gadermaier G. Do plantago lanceolata skin prick test-positive patients display IgE to genuine plantain pollen allergens? investigation of pollen allergic patients from the north-East of France. Int Arch Allergy Immunol (2018) 177:97–106. doi: 10.1159/000490004 29936506

[30] Buendia E Zakzuk J Mercado D Alvarez A Caraballo L. The IgE response to ascaris molecular components is associated with clinical indicators of asthma severity. World Allergy Organ J (2015) 8:8. doi: 10.1186/s40413-015-0058-z 25780492 PMC4347909

[31] Acevedo N Mohr J Zakzuk J Samonig M Briza P Erler A. Proteomic and immunochemical characterization of glutathione transferase as a new allergen of the nematode ascaris lumbricoides. PloS One (2013) 8:e78353. doi: 10.1371/journal.pone.0078353 24223794 PMC3817249

[32] Acevedo N Sanchez J Erler A Mercado D Briza P Kennedy M. IgE cross-reactivity between ascaris and domestic mite allergens: the role of tropomyosin and the nematode polyprotein ABA-1. Allergy. (2009) 64:1635–43. doi: 10.1111/j.1398-9995.2009.02084.x 19624559

[33] Caraballo L Puerta L Jimenez S Martinez B Mercado D Avjiouglu A. Cloning and IgE binding of a recombinant allergen from the mite blomia tropicalis, homologous with fatty acid-binding proteins. Int Arch Allergy Immunol (1997) 112:341–7. doi: 10.1159/000237478 9104789

[34] Zakzuk J Jimenez S Cheong N Puerta L Lee BW Chua KY. Immunological characterization of a blo t 12 isoallergen: identification of immunoglobulin e epitopes. Clin Exp Allergy (2009) 39:608–16. doi: 10.1111/j.1365-2222.2008.03193.x 19226278

[35] van Gemert F van der Molen T Jones R Chavannes N. The impact of asthma and COPD in sub-Saharan Africa. Prim Care Respir J (2011) 20:240–8. doi: 10.4104/pcrj.2011.00027 PMC6549843 21509418

[36] Quanjer PH Stanojevic S Cole TJ Baur X Hall GL Culver BH. Multi-ethnic reference values for spirometry for the 3-95-yr age range: the global lung function 2012 equations. Eur Respir J (2012) 40:1324–43. doi: 10.1183/09031936.00080312 PMC3786581 22743675

[37] Niespodziana K Stenberg-Hammar K Papadopoulos NG Focke-Tejkl M Errhalt P Konradsen JR. Microarray technology may reveal the contribution of allergen exposure and rhinovirus infections as possible triggers for acute wheezing attacks in preschool children. Viruses. (2021) 13:915. doi: 10.3390/v13050915 34063445 PMC8155838

[38] Sibanda EN. Inhalant allergies in Zimbabwe: a common problem. Int Arch Allergy Immunol (2003) 130:2–9. doi: 10.1159/000068377 12576728

[39] Rujeni N Nausch N Bourke CD Midzi N Mduluza T Taylor DW. Atopy is inversely related to schistosome infection intensity: a comparative study in Zimbabwean villages with distinct levels of schistosoma haematobium infection. Int Arch Allergy Immunol (2012) 158:288–98. doi: 10.1159/000332949 PMC3398828 22398631

[40] Van den Biggelaar AH Lopuhaa C van Ree R van der Zee JS Jans J Hoek A. The prevalence of parasite infestation and house dust mite sensitization in gabonese schoolchildren. Int Arch Allergy Immunol (2001) 126:231–8. doi: 10.1159/000049519 11752881

[41] Lule SA Mpairwe H Nampijja M Akello F Kabagenyi J Namara B. Life-course of atopy and allergy-related disease events in tropical sub-Saharan Africa: a birth cohort study. Pediatr Allergy Immunol (2017) 28:377−83. doi: 10.1111/pai.12719 28339128 PMC5488189

[42] Kim CR Jeong KY Yi MH Kim HP Shin HJ Yong TS. Cross-reactivity between group-5 and -21 mite allergens from dermatophagoides farina, tyrophagus putrescentia and blomia tropicalis. Mol Med Rep (2015) 12:5467–74. doi: 10.3892/mmr.2015.4093 26238285

[43] Reginald K Pang SL Chew FT. Blo t 2: group 2 allergen from the dust mite blomia tropicalis. Sci Rep (2019) 9:12239. doi: 10.1038/s41598-019-48688-y 31439916 PMC6706440

[44] D'Souza N Weber M Sarzsinszky E Vrtala S Curin M Schaar M. The molecular allergen recognition profile in China as basis for allergen-specific immunotherapy. Front Immunol (2021) 12:719573. doi: 10.3389/fimmu.2021.719573 34512644 PMC8430339

[45] Mourao EMM Rosario NA. Conjunctival provocation test with blomia tropicalis. Front Allergy (2021) 2:673462. doi: 10.3389/falgy.2021.673462 35386969 PMC8974718

[46] Yi FC Chew FT Jimenez S Chua KY Lee BW. Culture of blomia tropicalis and IgE immunoblot characterization of its allergenicity. Asian Pac J Allergy Immunol (1999) 17:189–94. 10697258

[47] Zakzuk J Mercado D Bornacelly A Sanchez J Ahumada V Acevedo N. Hygienic conditions influence sensitization to blomia tropicalis allergenic components: results from the FRAAT birth cohort. Pediatr Allergy Immunol (2019) 30:172–8. doi: 10.1111/pai.13004 30421833

[48] Chew FT Zhang L Ho TM Lee BW. House dust mite fauna of tropical Singapore. Clin Exp Allergy (1999) 29:201–6. doi: 10.1046/j.1365-2222.1999.00493.x 10051724

[49] Amini P Abdullah M Seng LS Karunakaran T Hani N Bakar SA. Ethnicity influences disease characteristics and symptom severity in allergic rhinitis patients in Malaysia. Int Forum Allergy Rhinol (2016) 6:624–30. doi: 10.1002/alr.21442 26919193

[50] Munera M Martinez D Labrada A Caraballo L Puerta L. Identification of B cell epitopes of Blo t 3 allergen and cross-reactivity with human adipocytes and heart fatty acid binding proteins. Int J Mol Sci (2019) 20:6107. doi: 10.3390/ijms20246107 31817065 PMC6940925

[51] Santos da Silva E Marques Ponte JC Barbosa da Silva M Silva Pinheiro C Carvalho Pacheco LG Ferreira F. Proteomic analysis reveals allergen variability among breeds of the dust mite blomia tropicalis. Int Arch Allergy Immunol (2019) 180:159–72. doi: 10.1159/000501964 31563904

[52] Resch Y Michel S Kabesch M Lupinek C Valenta R Vrtala S. Different IgE recognition of mite allergen components in asthmatic and nonasthmatic children. J Allergy Clin Immunol (2015) 136:1083–91. doi: 10.1016/j.jaci.2015.03.024 PMC4595482 25956509

[53] Vaerenbergh M Cardoen D Formesyn EM Brunain M Van Driessche G Blank S. Extending the honey bee venome with the antimicrobial peptide apidaecin and a protein resembling wasp antigen 5. Insect Mol Biol (2013) 22:199–210. doi: 10.1111/imb.12013 23350689

[54] Cabauatan CR Lupinek C Scheiblhofer S Weiss R Focke-Tejkl M Bhalla PL. Allergen microarray detects high prevalence of asymptomatic IgE sensitizations to tropical pollen-derived carbohydrates. J Allergy Clin Immunol (2014) 133:910–4. doi: 10.1016/j.jaci.2013.10.004 PMC6597356 24315449

[55] Casset A Mari A Purohit A Resch Y Weghofer M Ferrara R. Varying allergen composition and content affects the *in vivo* allergenic activity of commercial Dermatophagoides pteronyssinus extracts. Int Arch Allergy Immunol (2012) 159:253–62. doi: 10.1159/000337654 PMC4594775 22722650

[56] Westritschnig K Horak F Swoboda I Balic N Spitzauer S Kundi M. Different allergenic activity of grass pollen allergens revealed by skin testing. Eur J Clin Invest (2008) 38:260–7. doi: 10.1111/j.1365-2362.2008.01938.x 18339006

[57] Alcantara-Neves NM Veiga RV Ponte JC da Cunha SS Simoes SM Cruz AA. Dissociation between skin test reactivity and anti-aeroallergen IgE: determinants among urban Brazilian children. PloS One (2017) 12:e0174089. doi: 10.1371/journal.pone.0174089 28350867 PMC5369757

[58] Caraballo L Valenta R Acevedo N Zakzuk J. Are the terms major and minor allergens useful for precision allergology? Front Immunol (2021) 12:651500. doi: 10.3389/fimmu.2021.651500 33763086 PMC7982392

[59] Cardona-Villa R Uribe-Garcia S Calvo-Betancur VD Cantillo JF Fernandez-Caldas E. Efficacy and safety of subcutaneous immunotherapy with a mixture of glutaraldehyde-modified extracts of dermatophagoides pteronyssinus, dermatophagoides farinae , and blomia tropicalis. World Allergy Organ J (2022) 15:100692. doi: 10.1016/j.waojou.2022.100692 36119655 PMC9467880

[60] Castro-Almarales RL Ronquillo-Diaz M Alvarez-Castello M Rodriguez-Canosa J Gonzalez-Leon M Enriquez-Dominguez I. Subcutaneous allergen immunotherapy for asthma: a randomized, double-blind, placebo-controlled study with a standardized blomia tropicalis vaccine. World Allergy Organ J (2020) 13:100098. doi: 10.1016/j.waojou.2020.100098 32308779 PMC7155230

[61] Barboza R Camara NO Gomes E Sa-Nunes A Florsheim E Mirotti L. Endotoxin exposure during sensitization to blomia tropicalis allergens shifts TH2 immunity towards a TH17-mediated airway neutrophilic inflammation: role of TLR4 and TLR2. PloS One (2013) 8:e67115. doi: 10.1371/journal.pone.0067115 23805294 PMC3689683

[62] Labrada A Facenda E Castro RL Fernandez B Uyema K Sewer M. State of the art in developing allergen vaccines in Cuba: prospects of novel adjuvanted vaccines. Vaccine. (2006) 24 Suppl 2:S2–36-7. doi: 10.1016/j.vaccine.2005.01.111 16823917

